# Norm and Deviance-Seeking Personal Orientation Scale (NDPOS) Adapted to the Organisational Context

**DOI:** 10.5334/pb.462

**Published:** 2019-10-21

**Authors:** Guillaume Roland Michel Déprez, Adalgisa Battistelli, Mirko Antino

**Affiliations:** 1 Université de Bordeaux, Laboratoire de psychologie EA4139, FR; 2 Dpto. Metodología de las Ciencias del Comportamiento, Facultad de Psicología, Universidad Complutense de Madrid, ES

**Keywords:** normativity, deviance, personal orientation, measure validity, measurement scale

## Abstract

Deviance theory introduces a behavioural view on constructive and destructive deviance to explain how an individual’s intent can harm or improve organisational well-being. However, to our knowledge, no scale exists that evaluates the personal orientation aspect of deviance and normativity. This article discusses the creation of the Norm and Deviance-Seeking Personal Orientation Scale (NDPOS). To create this scale, we studied the psychometric properties of the instrument using data from French workers. NDPOS exploratory analysis indicated a 12-item scale composed of four factors: normative conformity, normative rule adequacy, deviant performance seeking, and deviant proactivity seeking. Confirmatory factor analysis corroborated the factorial structure in four sub-scales. Convergent and discriminant validity indicated that deviant dimensions were positively related to expressing voice, cognitive flexibility, and deviant behaviours, whereas normativity dimensions were negatively or not related to these behaviours. Furthermore, opposite relations between the conformity construct and the four factors were observed. Practical implications and suggestions for the development of future research on constructive deviance theory are discussed.

## Introduction

The theme of deviance in work and organisational psychology has been extensively studied (e.g. Bennett & Robinson, 2000; Spreitzer & Sonenshein, 2004). Normative approaches focus on intentional departure from the norm of the group or organisation in which the individual develops and they are related to the underlying intention of deviation (Spreitzer & Sonenshein, 2004). In most cases, researches focus on the positive or negative, behavioural contribution of the deviant and normative processes (see Vadera, Pratt, & Mishra, 2013). Indeed, the normative and deviant constructs are generally addressed by studying their behavioural dimensions (Galperin, 2012; Robinson & Bennett, 1995; Vadera et al., 2013), neglecting the personal orientation aspect. The latter aspect should be further studied, as it would make it possible to analyse the processes underlying the emergence of deviant, change, and innovative behaviours; however, this is not possible with the current behavioural approaches. In the current climate of change and innovation, it is essential to determine which factors facilitate the implementation of change and innovative behaviours (be they behavioural, attitudinal, contextual, etc.), and whether change and innovative constructs are similar (Potočnik & Anderson, 2016). The factors relating to deviant and normative personal orientation should be studied to provide a better understanding of the intentional process that leads to behavioural choices. Thus, a scale evaluating these different orientations is essential because it will allow determining the role played by deviance and normativity in shaping processes of change and innovation. However, no study adapted to the workplace offers tools for assessing normativity and deviance from an orientation perspective.

In this study, we make several contributions to the literature. Foremost, we developed and tested the Norm and Deviance-Seeking Personal Orientation Scale (NDPOS), adapted to the organisational context. To do this, we conducted a brief review of the literature and proposed a personal orientation approach to evaluate deviance and normativity at work based on the active minorities’ theories (Moscovici, 1979, 1984) and the constructive deviant theories (Warren, 2003). Then, by carrying out three studies allowing the creation of the items and analysis of the scale’s validity and its measurement invariance, we tested the psychometric qualities of the developed scale, showing the necessity of using personal orientation with behavioural approaches to better understand deviance and normativity at work. Finally, we discussed all the results according to the literature and proposed possible uses for the NDPOS.

### Towards the Normative- and Deviance-Seeking Personal Orientation Approach

Both deviance and normative conceptualisations encompass specifics behaviours (e.g. Cropanzano, Anthony, Daniels, & Hall, 2017; Vadera, Pratt, & Mishra, 2013). However, these conceptualisations do not consider the personal orientation aspect of the constructs. Dahling and Gutworth (2017) recently pointed out the need to develop a personality approach to properly investigate the relationships between normative conflict, psychological discomfort, and constructive deviance. Accordingly, we suggest using personal orientation, that is, an individual’s predisposition “to interpret, evaluate, and act on social reality in certain ways” (Wheaton, 1983: 210). It is perceived as an individual’s internal orientation that can lead to a behavioural realisation. Thus, it is necessary to investigate the orientation perspective of deviance or normativity (i.e., the general tendency of individuals regarding whether to try to respect rules, conform, optimise their performance, or take initiative, even if it means deviating from formal or informal norms).

Scholars generally approach the construct of deviance through two streams of research: the negative side (e.g. Judge, Scott, & Ilies, 2006) and the positive (e.g. Galperin, 2012). The negative side, labelled as destructive deviant behaviour, is defined as a ‘voluntary behaviour that violates significant organizational norms and in so doing threatens the well-being of an organization, its members, or both’ (Robinson & Bennett, 1995; p. 557). It includes several constructs from stealing to withdrawal (see Spector et al., 2006). The positive side is related to “voluntary behaviour that violates significant norms with the intent of improving the well-being of an organization, its members or both” (Galperin, 2002) and is defined as constructive deviance (for a review, see Mertens, Recker, Kummer, Kohlborn, & Viaene, 2016). It can be characterised by breaking the rules to help a co-worker (Dahling, Chau, Mayer, & Gregory, 2012). In studying deviance, it is also necessary to analyse the normative aspect (Warren, 2003). In management research, norms are defined as regular patterns which are relatively stable and expected by group members (Bettenhausen & Murnighan, 1991; p. 21). As mentioned by Warren (2003), in constructive deviance literature, it is common to refer to hypernorms (Donaldson & Dunfee, 1994, 1999) to avoid problems caused by informal and formal norms and the specificity of normality (Axelrod, 1986). For example, in the case where an entire company endorses informal norms that consciously break the law, illegal behaviours are perceived as normal (Brief, Buttram, & Durkeich, 2001) even though all the company members consciously violate the law. The reference to hypernorms makes it possible to refer to metanorms specific to human values and beliefs (Donaldson & Dunfee, 1999; Sherif, 1936) to determine positive or negative behaviours, whether normal or deviant. Departing from the behavioural approach specific to the study of deviance and normativity, personal orientation will promote a deeper understanding allowing better understanding of the emergence of behaviours labelled as deviant or normative, and their interaction with contextual variables. For example, in an organisational setting where job characteristics are changing, the use of personal orientation contextualisation will allow to capture the emergence of constructive deviance or voice behaviour more completely, as it will permit study of the evolution from voice to constructive deviance, and vice versa, depending on the organisational climate’s nature. However, this dynamic and evolving aspect of deviance remains understudied.

Research on deviance and normativity requires to consider both the orientation towards an act and the behaviour itself (Bacon, Lenton-Maughan, & May, 2018) and how one can lead to the other (Creed et al., 2017). Thus, Spreitzer and Sonenshein (2003) introduce the notion of group norms and the specific intention to detach oneself from them in an honourable way. They note the importance of distinguishing deviant from honourable and virtuous intent, as virtuous intent is in opposition to the principle of deviance (Peterson & Seligman, 2003). Whether one’s intent is honourable and virtuous thus depends on an individual’s perception and working context, and breaking his or her organisational or proximal norms remains a key factor in a worker’s choice to resolve an issue in a normative or deviant manner (Jetten & Hornsey, 2014), both for constructive and destructive intent (Acharya & Taylor, 2012; Warren, 2003). Accordingly, deviance cannot be considered an isolated tendency, or only an intention, but rather a transitory personal orientation state, depending on the surrounding context, which would lead to the establishment of behaviours (Lee, Park, & Koo, 2015). In this perspective, personal orientation to deviance and normativity contributes more than constructs such as prosocial rule-breaking (PSRB), constructive deviance behaviour, and constructive voice behaviour. Indeed, where constructs such as PSRB or voice can capture behaviour and intention (Dahling et al., 2012; Morrison, 2006), personal orientation makes it possible to extract individual trends (Wheaton, 1983) and thereby accurately target the mechanism underlying their development. For example, the study of deviant and normative personal orientation will allow determining the emergence or inhibition of intention or behaviour to break established rules in the context of change and innovation. Using theories from social psychology on the theme of active minorities (e.g. Levine & Zdaniuk, 1984; Moscovici & Mugny, 1987), we thus focus on the relationship between normative and deviant personal orientation that does not imply a notion of good or evil, and therefore does not refer to hypernorms. We believe this is relevant, as personal orientation allows us to capture ‘the individual’s general style of behaving to meet underlying personal needs’ (Creed, Hood, & Hu, 2017).

The study of deviant and normative personal orientation is thus necessary for at least three reasons. First, the literature on deviance and normativity presents them as two constructs playing a decisive role in organisational change (Galperin, 2012). In most cases, research studying the organisational change process focuses on the generation of deviant behaviours (Spector et al., 2006) and neglects the role played by deviant and normative personal orientation. It is essential to analyse the role played by personal orientation in the development of behavioural responses to change to predict their progress. Indeed, personal orientation can lead to behaviours generating positive or negative organisational change (Shin, Taylor, & Seo, 2012). Second, it is necessary to overcome the emphasis on positive or negative behaviours to highlight the essence of deviance and normativity. The strong positive relationship between constructive and destructive deviance signals an underlying process (Warren, 2003) which we might attribute to the personal orientation dimension. This can explain the existing relationship between proactive, prosocial, and deviant behaviours, although these constructs belong to different organisational realities (Vadera et al., 2013). The orientation perspective on deviance and normativity supposes the existence of a neutral state which can affect whether positive or negative behaviours emerge, depending on organisational, contextual, and individual factors. Finally, the study of attitudes in organisations provides key information for companies concerning the performance and behaviour of their employees (O’Neill & Hasting, 2011). Indeed, the study of the orientation-behaviour relationship makes it possible to understand organisational processes more clearly and apprehend the emergence of behaviours globally. For example, in an organisational change situation in which conflicts are present, it will allow determining whether deviant or proactive behaviours can emerge.

Regardless of whether studies have examined the normative aspect, few have used scales for their research (Reysen & Branscombe, 2008), preferring experimental procedures (e.g. Asch, 1956; Hackman, 1992; Moscovici, 1979), and only scales with behavioural dimensions have been developed for the deviant dimension (e.g. Dahling et al., 2012; Spector et al., 2006). The aim of this research is to develop a scale adapted to the organisational context which can be used by both scientists and practitioners. Although an inclination towards the experimental method is growing in the organisational context (Hauser, Linos, & Rogers, 2017), the use of a scale of measurement will remain a significant tool. Indeed, being able to evaluate the personal orientation of each individual towards deviance or conformity will allow to better target of potentially emergent behavioural responses. It will also allow evaluation, by a self-reported method, of individuals’ perception of their personal orientation towards deviance and normativity. Thus, we created the NDPOS, a four-factor scale composed of two normative and two deviant dimensions (see Table 1). Concerning the normative aspect, the existence of two factors (*conformity* and *rules adequacy*) fit the organisational context. Normativity gathers formal and informal norms; however, the line between these two terms is sometimes unclear (Edgerton, 1985). The dissociation between *conformity* and *rule adequacy* is intended to avoid any confusion between informal and formal norms. The orientation to conformity has been studied extensively and ‘occurs when an individual modifies his attitude to bring it more in line with the attitude of a group’ (e.g. Crutchfield, 1955; Moscovici, 1984). This personal orientation corresponds to the informal aspect of normativity, given its objective of generating behaviours expected or tolerated by the social group (Axelrod, 1986; Moscovici, 1979). The personal orientation of rules adequacy is related to the propensity of individuals to respect, or not respect, organisational guidelines (Morrison, 2006). Therefore, this personal orientation is related to the formal aspect of normativity, given its goals to fit to “explicit organizationally defined policy, regulation, or prohibition” (Morrison, 2006; p. 6). Each of the dimensions of the normative construct was a component of the normative dimension. Regarding the deviant dimension, the two factors chosen were *performance seeking* and *proactivity seeking*, which can both lead to destructive and/or constructive outcomes (Galperin, 2002; Warren, 2003). Some of the reasons that lead people to deviate from norms and rules are their propensity to try to be more performant (Morrison, 2006) or to reduce normative conflict by acting in advance (Dahling & Gutworth, 2017; Vadera et al., 2013). We argued that performance seeking is related to the first orientation and proactivity seeking to the second. Indeed, orientation towards deviant performance seeking refers to the propensity of the individual to seek efficiency and better performance with no care for the respect of rules or norms (see Mertens et al., 2016). Whereas, orientation towards deviant proactivity seeking refers to the individual tendency of the worker to try to act in advance of events with no regard for rules. Thus, although these two dimensions do not allow us to encompass all the deviant personal orientation process, they do allow us to target two essential parts of it (Galperin, 2002; Spreitzer & Sonenshein, 2004; Warren, 2003). Our literature review seems to highlight the importance of studying and developing a scale of measurement regarding deviance and normativity in view of an approach centred on personal orientation.

**Table 1:** Types of Orientations, Specifics Dimensions, Construct Definitions, and Illustrative Personal Orientations.

Orientations	Dimensions	Definitions	Examples

*Normativity*		*Individual propensity to develop an orientation to conform and/or following the norm, policies, or rules*	
	Conformity	Ensure that his/her personal orientation matches that of the group.	Tend to conform to other’s choice rather than have an opinion.
	Rule Adequacy	Ensure that his/her personal orientation matches the established rules.	Tend to follow the rules in any situation, even if it seems pointless.
*Deviance*		*Individual propensity to develop an orientation to deviate from the norm, policies, or rules*	
	Performance seeking	Deviant personal orientation towards the search for efficiency and/or effectiveness.	Tend to break some rules, norms, or stereotypes to be more efficient.
	Proactivity seeking	Deviant personal orientation towards proactivity, prevention, and improvement of the surrounding context.	Tend to deviate from norms to prevent potential discomfort.

To test the psychometric qualities of NDPOS, we followed a multiphase analysis process (Hinkin, 1995) composed of three studies using different samples. In the first study, to generate items, a review of the theory and measures of deviance and normativity was conducted. Then, each generated item was submitted for expert evaluation to assess its theoretical significance. Based on expert evaluation of the items, we tested the item pool reliability results and internal scale consistency, and we used exploratory factor analysis (EFA) to uncover the underlying structure of the final NDPOS. In the second study, to confirm the structure of the overall scale, we conducted a confirmatory factor analysis (CFA) on the four-factor structure and analysed the factors’ relationships with similar theoretical constructs by performing correlations and a CFA. Finally, to ensure that the factor scale did not vary depending on demographic factors, we conducted an invariance analysis. Analyses were performed using Mplus 8.1 (Muthen & Muthen, 2017).

## Study 1 Item Generation and Measure Development

Few studies have used a scale to test deviant and normative models (e.g. Dahling & Gutworth, 2017; Reysen & Branscombe, 2008), preferring experimental methods. Consequently, we needed to develop our measure of normativity and deviance concerning personal orientation.

### Methods

#### Procedure

According to recommended procedures (e.g. DeVellis, 1991, 2003), during the generation phase, we oversampled the number of items to separately capture the four domain constructs, thus ultimately reducing to three- to four-item sub-scales (Hinkin, 1995; Little, Lindenberger, & Nesselroade, 1999). We formulated a large pool of items (fifteen for each dimension) and asked experts in the field of work and organisational psychology to ensure the items’ clarity, redundancy, and adequate representation of the constructs. Based on the experts’ feedback, we deleted and modified several items and ultimately retained 20 from the initial pool (five items for each dimension).

#### Sample

Using the 20-item pool, we administered a questionnaire to employees (*N* = 311) recruited through a social network who worked either full- or part-time. The sample was essentially composed of women (88%) working in the private sector (50.8%). Each of the respondents were taking classes at universities in France, and 66% of them had completed a bachelor’s degree. The sample ranged in age from 18 to 55 years (*M* = 24.66, *SD* = 5.78), with a mean job tenure of 24.2 months. The anonymity of the participants was ensured. Respondents were asked to read each item carefully and indicate on a Likert-type scale from 1 (strongly disagree) to 5 (strongly agree) the extent to which they concurred that the items described their workplace personal orientation. An example of an item used was ‘I tend to use new organisational methods if they are approved by the company’.

### Results

To assess the factor structure, we used EFA (Thompson, 2004) using maximum likelihood method and geomin rotation. We opted to use an oblique rotation (geomin) according to our theoretical expectations that deviance dimensions and normativity dimensions were interrelated (Tabachnick & Fidell, 2012). To determine the number of factors, we followed Kline (2016) and Brown (2015) recommendations by using model comparison analysis (the Satora-Bentler chi-square, and the difference between CFI and TLI values) and model fit indices (the Root Mean Square Error of Approximation, the Chi-square value and its degree of freedom, Comparative Fit Index, the Tucker-Lewis Index, the Standardized Root Mean Square Residual, the Akaike Information Criterion, and the Bayesian Criteria Information) provided by Mplus 8.1. Internal consistency was measured using model fit indices analysis and Cronbach’s alpha. Item analysis indicated that seven items had a total scale factorial correlation of less than .30. Following Nunnally and Bernstein’s (1994) recommendations, these items were deleted. Based on these analyses, 12 items remained. The expected dimensionality was confirmed by the results of the EFA, indicating a four-factor solution (χ^2^ (24) = 31.11, *p* > .05; RMSEA = .03; CFI = .99; TLI = .97; SRMR = .01; AIC = 10218.320; BIC = 10465.147) with eigenvalues from 4.17 to 1.07, and a total explained variance of 63.37% (see Table 2). The four-factor solution outperformed models such as a three-factor solution (χ^2^ (33) = 127.99, p < .001; RMSEA = .09; CFI = .89; TLI = .78; SRMR = .04; AIC = 10289.252; BIC = 10502.420; Δχ^2^ = 208.04, df = 19, p < .01; ΔCFI = .10; ΔTLI = .19), a two-factor solution (χ^2^ (43) = 237.11, p < .001; RMSEA = .12; CFI = .77; TLI = .66; SRMR = .06; AIC = 10377.465; BIC = 10553.235; Δχ^2^ = 208.04, df = 19, p < .01; ΔCFI = .22; ΔTLI = .31), and a one-factor solution (χ^2^ (54) = 351.688, p < .001; RMSEA = .13; CFI = .66; TLI = .58; SRMR = .09; AIC = 10521.722; BIC = 10656.355; Δχ^2^ = 292.86, df = 30, p < .01; ΔCFI = .33; ΔTLI = .39). A five-factor solution was tested but did not converge due to exceeding iterations.

**Table 2:** Norm and Deviance-seeking Personal Orientation Scale Items and Factor Loadings (N = 311).

Items	Factor			

	Normativity		Deviance	

	Conformity	Rule Adequacy	Performance Seeking	Proactivity Seeking

To ensure tranquillity, I prefer to conform to the group’s point of view. *Je préfère me conformer à l’avis du groupe pour assurer ma tranquillité*.	**.74**	–.08	–.02	–.08
I prefer to conform to the group’s choice whether I have an opinion or not on a matter. *Que j’ai ou non un avis sur n’importe quelle question je préfère me conformer au choix du groupe*.	**.54**	.02	–.02	–.11
I try to avoid conflicts by conforming to the group *J’essaie d’éviter des conflits possibles en me conformant au groupe*.	**.66**	.11	–.00	.07
I try to abide to my supervisor’s ways of doing things even though I find them inadequate *J’essaie d’utiliser des démarches définies par mon superviseur même si elles me paraissent inadaptées*.	–.01	**.74**	.06	–.12
I will try to follow an organizational rule, even if it seems pointless. *Si une règle organisationnelle me paraît inutile, je tente de l’appliquer tout de même*.	.02	**.66**	–.15	–.00
I try to conform to organizational decisions even if I disagree with them. *J’essaie de me conformer aux décisions organisationnelles même lorsque je suis en désaccord avec celles-ci*.	.03	**.67**	–.07	.06
I tend to break some organizational rules, in order to be more efficient. *J’ai tendance à transgresser certaines règles organisationnelles pour être plus efficace*.	–.03	.05	**.79**	.00
I do not hesitate to break some organizational rules when I perceive that they hinder my performance. *Je n’hésite pas à transgresser certaines règles organisationnelles lorsque j’estime qu’elles diminuent mon efficacité*.	.02	–.04	**.81**	–.03
I tend to break organizational rules that I find pointless *J’ai tendance à transgresser les règles organisationnelles qui me paraissent défaillantes*.	–.01	–.24	**.50**	.11
If I think there is a better way of doing things compared to what the group proposed, I am not shy of sharing my ideas. *Si j’estime que l’on peut agir différemment de ce qui est proposé par le groupe, j’essaie de le faire savoir*.	–.03	.01	–.00	**.86**
I try to tell my supervisors when I see shortcomings in the directions he gives me. *J’essaie de faire part à mon superviseur des défaillances que je perçois dans les consignes qu’il me donne*.	–.01	–.02	–.00	**.49**
I try to bring new work practices that have not been used by my colleagues. *J’essaie d’apporter de nouvelles pratiques de travail non utilisées par mes collègues*.	.03	–.12	.11	**.38**
Eigenvalues	4.05	1.50	1.34	1.07
% variance explained	32.17	12.23	10.76	8.21

*Note*: Primary loadings are in bold. All items were administered in French, English translations for communication purposes.

In our interpretation, the first and second factors were subscales of the normativity dimension, and the third and fourth factors were subscales of the deviance dimension. The first factor reflected orientation to conformity; the second, the inclination to respect rules; the third, the tendency to deviate from norms and rules for efficiency; and the fourth, the propensity to try to deviate from taking initiative. Three of these subscales showed adequate reliability (Table 3), and only the deviant proactivity seeking dimension had a lower score (α = .61). Although the conventional accepted minimum is .70 (see Peterson, 1994), the lower limit can decrease to .60 in exploratory analysis (Hair et al., 2010). Furthermore, as specified by Briggs & Cheek (1986), Cortina (1993), and Field, Miles, & Field (2012), when there are a small number of items in a scale (<10), Cronbach’s alpha values can be quite low. In this situation, it is better to calculate the items’ mean inter-item correlation. Optimal mean inter-item correlation values range from .2 to .4 (Briggs & Cheek 1986). Concerning the proactivity seeking dimension, we obtained adequate inter-item correlation values (.23 to .40).

**Table 3:** Factor Correlation Matrix, Mean, Standard Deviations, and Reliabilities (N = 311).

		*M*	*SD*	1	2	3	4

1.	Conformity	3.07	.78	*(.71)*			
2.	Rule adequacy	2.95	.87	.50**	*(.77)*		
3.	Performance seeking	2.98	.96	–.20**	–.40**	*(.78)*	
4.	Proactivity seeking	3.76	.78	–.33**	–.26**	.25**	*(.61)*

*Note*: ** *p* < .01; Number in parentheses are the Cronbach’s alpha scores.

### Discussion

Study 1 concluded with a 12-item factorial structure. The NDPOS was composed of four potential subscales related to normative and deviant personal orientation. Results suggested that the scale had appropriate validity (Hinkin, 1995). Following Hinkin’s (1995) recommendations, a new sample was collected to confirm the factor structure and provide initial evidence of the discriminant and criterion-related validity of the scale. As specified by Brown (2015), “CFA is an indispensable analytic tool for construct validation in the social and behavioural sciences.” (p. 2).

## Study 2 Structure Validation and Validity Assessment

Study 2 was conducted to confirm the factor structure of the NDPOS, using CFA, and to validate the NDPOS relative to related and unrelated dimensions. To assess convergent and discriminant validity, we performed a correlation analysis between the NDPOS dimensions and specific constructs.

### Hypothesis Development

In the absence of much existing research on normative and deviant personal orientations in the organisational context, we focused our attention on the literature on positive deviance behaviours. Thus, we chose to study the positive and negative relationship with constructs such as conformity and cognitive flexibility. Moreover, to dissociate our four factors from behavioural constructs, we compared them to the behaviour of voice, constructive deviance, and PSRB. Indeed, they are presented as essential deviant constructs (Vadera et al., 2013). Exploring the relationship between chosen constructs and the NDPOS factors would thus inform us about the scale’s validity.

We first proposed examining conformity, which occurs when an individual modifies his/her attitude to bring it more in line with the attitude of a group (Levine & Zdaniuk, 1984). According to the literature, conformity is not the default value in a group, nor is deviance and dissidence the exception (Jetten & Hornsey, 2014). Thus, conformity could be observed under some specific situations (Deutsch & Gerard, 1955), such as facing uncertainty or gaining approval from others. To reduce uncertainty or be approved of by others, individuals must respect group rules and show identification with group values (Jetten & Hornsey, 2014). Personal orientations to conformity and rules adequacy are constructs that allow us to respect group normativity, whereas deviant constructs are characterised by an orientation to go against group rules and identified norms (Moscovici & Mugny, 1987; Warren, 2003). Accordingly, we formulated the following hypothesis:

Hypothesis 1: Conformity will be positively related to NDPOS normative dimensions and negatively related to NDPOS deviant dimensions.

The second construct we examined was cognitive flexibility, which is defined as ‘the essential ability to assess and adapt ongoing psychological operations and to coordinate the allocation of cognitive processes appropriately in dynamic decision-making environments’ (Glass, Madox, & Love, 2013; p. 2). Individuals with high levels of flexibility are more attentive, perspicacious, and receptive, and more capable of testing new methods of social interactions (Martin & Anderson, 1998). High levels of cognitive flexibility predict a good capacity to consider problems or solutions under new views, which facilitates the generation of alternative ideas (Binard & Pohl, 2014; Thurston & Runco, 1997). This propensity to consider new ways of thinking is characteristic of cognitive flexibility (Martin & Ruben, 1995). As the personal orientation to conformity is defined as a propensity to seek the adoption of rules, values, and group behaviours, we expected it to be lower when cognitive flexibility was high. Indeed, cognitive flexibility presents characteristics related to change and innovation (Binard & Pohl, 2014), which should not be the case for orientation to conformity, or the conformity behaviour presented by Reysen and Branscombe (2008). The personal orientation to rules adequacy, which is according to its normative nature closely related to personal orientation to conformity and following similar processes, should be lower when cognitive flexibility is high. Concerning the deviant aspect of the NDPOS, cognitive flexibility is related to deviant behaviours by the tendency to consider possible alternative actions (Martin & Ruben, 1995), and thus act in ways that depart from the norm to be more constructive. Accordingly, we hypothesised that the relationship between the two NDPOS deviant factors and cognitive flexibility would be positive.

Hypothesis 2: Cognitive flexibility will be negatively related to NDPOS normative dimensions and positively related to NDPOS deviant dimensions.

PSRB (Dahling et al., 2012) and constructive deviance (Galperin, 2012) are constructs of positive deviance (Morrison, 2006; Spreitzer & Sonenshein, 2004). The first behaviour takes three deviant organisational paths: (1) completing a task more efficiently, (2) aiding another employee, and (3) providing better customer service. The second behaviour is composed of two factors: interpersonal deviance (oriented to interaction with co-workers, customers, and/or one’s supervisor), and organisational constructive deviance (specific to the violating behaviours directed towards the organisational context). Warren (2003) argues that deviant intention should be related to the act of going against established rules, and to challenging specific existing norms. PSRB, by its rule-breaking process, and constructive deviance, by its norm-challenging process, should be positively related to the two deviant personal orientation factors (seeking performance and proactivity) and negatively related to the two normative factors (conformity and rules adequacy). We expected the relationship to highlight the disparity between personal orientation and behavioural paths and indicate whether there was an orientation towards deviance.

Hypothesis 3: PSRB will be negatively related to NDPOS normative dimensions and positively related to NDPOS deviant dimensions.

Hypothesis 4: Constructive deviant behaviour will be negatively related to NDPOS normative dimensions and positively related to NDPOS deviant dimensions.

Voice behaviour was the last proposed variable that we used to explore the relationship with the NDPOS. Voice behaviour can be driven by two different intents (Maynes & Podsakoff, 2014): promotive forms (supportive and constructive aspects), and prohibitive forms (defensive and destructive aspects). It is perceived as a proactive act (Parker & Collins, 2010) to speak about an event and share opinions regarding it (Van Dyne & Lepine, 1998). In certain circumstances, voice behaviour refers to the need to depart from the norms (Vadera et al., 2013) and induce change and innovation (Potočnik & Anderson, 2016). Accordingly, we proposed two relationships: a positive one with deviant orientation and a negative one with normativity, which implied more discretionary behaviours. However, the theoretical non-polarity of the NDPOS also suggested a positive link with the prohibitive forms of voice behaviours. Studying the relationship between voice behaviours and NDPOS factors allowed us to investigate whether personal orientation towards deviance and normativity were related to behaviours other than deviant constructs. Thus, we proposed the following:

Hypothesis 5: Voice behaviours will be negatively related to NDPOS normative dimensions and positively related to NDPOS deviant dimensions.

### Methods

#### Sample

The sample (*N* = 304) was composed of workers from different organisations and different sectors such as health (26%), social (22%), administration (18%), commerce (17%), research (11%), and industry (6%). Most respondents were women (84%) working in the private sector (51%), with an average organisational tenure of four years and an average age of 35 years (*SD* = 11). Some of the participants were supervisors (32%), and most worked in several teams (89%). The final version of the NDPOS used the same instructions and a five-point Likert-type scale similar to the one used in Study 1.

#### Measure

*Conformity* was measured using a three-item scale adapted from Reysen and Branscombe (2008). The items were designed to evaluate general conformity. The original version (OV) and the version of our article (FV) had acceptable Cronbach’s alphas (OV, α = .88; FV, α = .78). An example of an item used was ‘I generally conform to the norms of the groups to which I belong’.

*Cognitive flexibility* was assessed using a 12-item scale, originally created by Martin and Ruben (1995). For this study, we used the French translation (OV, α = .72; FV, α = .70) developed by Binard and Pohl (2014). Item examples included ‘I can communicate an idea in many different ways’ and ‘I am willing to work at creative solutions to problems’.

*PSRB* was evaluated by Dahling et al.’s (2012) 13-item measure, which included studying workers’ rule-breaking behaviours to complete a task more efficiently (OV, α = .89; FV, α = .75), help a co-worker (OV, α = .92; FV, α = .84), and help a customer (OV, α = .93; FV, α = .82). The French measure was composed of 11 items (Déprez, 2017). An example of an item used for the efficiency dimension was ‘I violate organisational policies to save the company time and money’; an example used regarding helping a co-worker was ‘I break organisational rules if my co-workers need help with their duties’; and an example used with respect to helping customers was ‘I break organisational rules to provide better customer service’.

*Constructive deviance* was assessed using Galperin’s (2012) measure, which analyses behaviours which violate norms towards the goal of producing constructive improvements at interpersonal and organisational levels. For this study, we used the French seven-item scale version (Déprez, 2017). Items used regarding the interpersonal dimension (OV, α = .67; FV, α = .67) were ‘[You] disobeyed your supervisor’s instructions to perform more efficiently’ and ‘[You] bent a rule to satisfy a customer’s needs’ for the organisational aspect (OV, α = .88; FV, α = .82). The items were evaluated with a five-point Likert-type scale from 1 (never) to 5 (daily).

*Voice* was evaluated via the 20-item measure from Maynes and Podsakoff (2014), which encompasses four dimensions of voice behaviours—two with a promotion orientation (supportive and constructive factors), and two with an inhibition orientation (defensive and destructive factors). Each of these dimensions was composed of five items: the constructive aspect (e.g. ‘Often suggests changes to work projects to make them better’; OV, α = .95; FV, α = .88), the supportive aspect (e.g. ‘Defends organisational programs that are worthwhile when others unfairly criticise the programs’; OV, α = .89; FV, α = .83), the defensive aspect (e.g. ‘Speaks out against changing work policies, even when making changes would be for the best’; OV, α = .92; FV, α = .73), and the destructive aspect (e.g. ‘Often makes overly critical comments about the organisation’s work practices or methods’; OV, α = .93; FV, α = .76).

### Results

#### Structure Validation

A CFA was performed to examine the four-factor structure of the 12-item scale obtained in the EFA analysis. Data were approximatively normally distributed, and thus, we used robust maximum likelihood (MLR) estimation. However, the kurtosis and skewness were within an absolute value of 2 (Tabachnik & Fidell, 2012). To assess internal validity, we tested the 12-item four-factor structure obtained by the EFA from Study 2 and compared it with four alternative models (Table 4). The first alternative model (1) was composed of three factors in which the performance seeking and proactivity seeking subscales were split and the normativity dimensions composed one unique factor. The second (2) integrated the two deviant factors as one, and the conformity and rule-adequacy factors were kept separate. The third (3) was composed of two factors in which rule adequacy and conformity were combined into one normative factor, and performance seeking and proactivity seeking were combined into one deviant factor. Finally, the fourth (4) was a one-factor model composed of all items of the four subscales.

**Table 4:** Fit statistic of the Initial and Alternative Models.

Model	χ^2^	*df*	RMSEA *(<.08)	RMSEA 90% CI	CFI *(>.9)	TLI *(>.9)	SRMR *(<.08)	AIC	BIC	Model comparison	ΔCFI	ΔTLI

Initial	95.091	48	.057	.040, .073	.965	.951	.038	8914.76	9070.87			
Model (1)	231.14	51	.108	.094, .122	.865	.825	.077	9057.19	9202.16	1 versus initial	–.100	–.126
Model (2)	261.40	51	.116	.842, .796	.842	.796	.090	9098.12	9243.08	2 versus 1	–.023	–.029
Model (3)	383.69	53	.143	.130, .157	.752	.691	.105	9222.59	9360.12	3 versus 2	–.090	–.105
Model (4)	741.13	54	.205	.192, .218	.484	.369	.174	9648.67	9782.48	4 versus 3	–.268	–.322

*Note*: *N* = 304. * *p* < .05; * cutoff.

**Figure 1:**
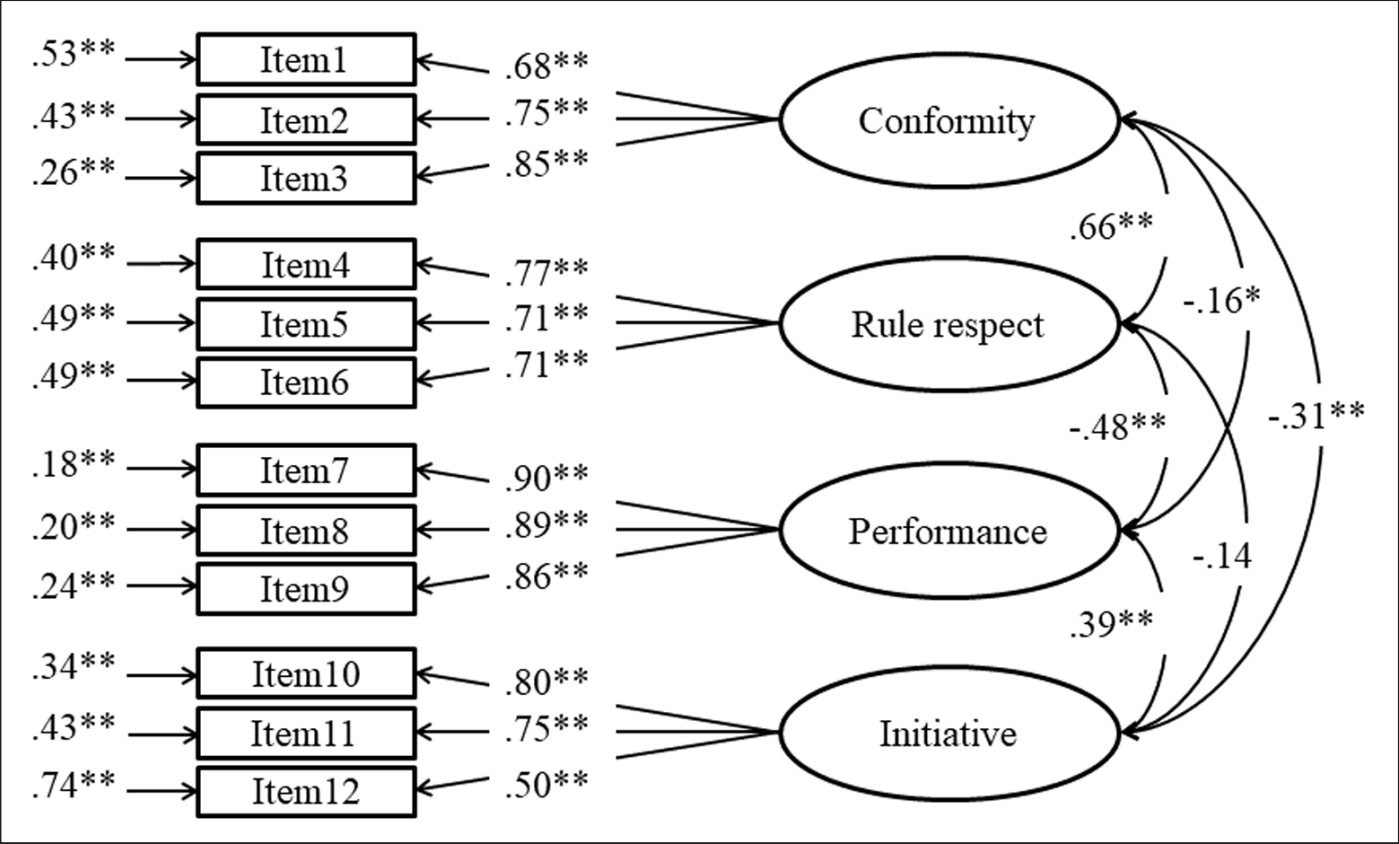
Confirmatory factor analysis of the NDPOS, study 3; * *p* < .05, ** *p* < .01.

The initial model fit indices were good (χ^2^ (48) = 95.091, *p* < .001; RMSEA = .05; CFI = .96; TLI = .95; SRMR = .03; AIC = 8914.763; BIC = 9070.878). None of the alternative models showed good fit indices, suggesting that the hypothesised model was the best. The standardised factor loadings, estimated factor correlations, and error variances of the initial model are displayed in Figure 1.

#### Validity Assessment: Test of Hypotheses

To test Hypotheses 1–5 and assess convergent and discriminant validity, we performed a correlation analysis between the NDPOS dimensions and conformity, cognitive flexibility, PSRB, constructive deviance, and voice. The results are presented in Table 5. As expected, the results with the conformity variable showed that the two deviant factors were negatively related to it, whereas the two normative factors were positively related to conformity. Only the performance-deviant seeking personal orientation was positively related to cognitive flexibility. Conformity-normative personal orientation was negatively related to cognitive flexibility. In the case of positive deviant behaviours (PSRB and constructive deviance), the performance-deviant seeking personal orientation showed the highest correlation (even with destructive deviance behaviour), higher than with the proactive seeking dimension of the NDPOS, which was more correlated with the voice promotion dimension. As expected, the two normative factors showed a negative correlation, or non-relation, with the voice dimensions and the positive deviant behaviours. To summarise, these results had good convergent and discriminant validities (Kline, 2016; McDowell & Newell, 2006), and the internal consistency of each scale was acceptable, specifically for the NDPOS (α = .72 to .91).

**Table 5:** Correlation among Deviant and Normative Orientation, and among Theoretical Correlate Behaviours.

		*M*	*SD*	1	2	3	4	5	6	7	8	9	10	11	12	13	14	15

1.	Deviant performance seeking	2.96	.98	(.91)														
2.	Deviant proactivity seeking	3.28	.72	.70**	(.72)													
3.	Normative conformity	2.64	.84	–.14*	–.16**	(.80)												
4.	Normative rule adequacy	2.73	.83	–.41**	–.24**	.53**	(.77)											
5.	Conformity	2.50	.84	–.19**	–.23**	.23**	.21**	(.78)										
6.	Cognitive flexibility	3.87	.44	.10	.22**	–.20**	–.09	–.35**	(.70)									
7.	PSRB efficiency	2.63	.94	.48**	.36**	–.03	–.27**	–.09	.12*	(.75)								
8.	PSRB co-worker	3.13	.98	.38**	.28**	–.11*	–.27**	–.05	.10	.57**	(.84)							
9.	PSRB customer	3.14	1.01	.45**	.34**	–.06	–.32**	–.08	.12*	.71**	.58**	(.82)						
10.	CDB interpersonal	2.50	.80	.48**	.37**	–.09	–.26**	–.09	.03	.45**	.44**	.42**	(.67)					
11.	CDB organizational	2.60	.82	.58**	.43**	–.18**	–.38**	–.10	.05	.49**	.44**	.48**	.78**	(.82)				
12.	Supportive voice	3.37	.77	.11*	.26**	–.10	–.00	–.13*	.17**	.07	.06	.01	.18**	.15**	(.83)			
13.	Constructive voice	3.42	.81	.18**	.39**	–.06	–.08	–.18**	.28**	.13*	.09	.08	.24**	.20**	.62**	(.88)		
14.	Destructive voice	1.88	.69	.33**	.24**	–.06	–.24**	–.07	–.10	.19**	.22**	.15**	.47**	.44**	–.00	.15**	(.76)	
15.	Defensive voice	1.85	.67	.10	.06	–.10	–.14*	–.09	–.12*	.00	.07	.02	.25**	.24**	.00	.08	.53**	(.73)

*Note*: *N* = 304; The Cronbach’s alpha corresponds to the number in brackets; * *p* < .05, ** *p* < .01; CDB = constructive deviance behaviour, PSRB = prosocial rule breaking.

### Discussion

The results in Study 2 were consistent with our four-factors structure expectations. We found evidence for the factorial stability and reliability of the NDPOS. Additionally, the positive correlations observed between the deviant personal orientation and the constructive deviant behaviours informed us about the existing relationship between the intentional and behavioural aspects of the constructs. Moreover, the correlation scores allowed us to verify that the NDPOS was indeed a personal orientation scale different from the behavioural measures (Kline, 2016). The observed positive relationship between voice (promotive and prohibitive dimensions) and the two normative orientations suggests that deviant personal orientation could be related to other constructs, in a positive or negative manner, as can be seen with the positive correlation scores between the two deviant orientations and the destructive voice factor. Only cognitive flexibility had a significant relationship with deviant orientation towards proactivity seeking. This could be because only the orientation towards proactivity seeking necessitates the ability to be cognitively flexible.

Concerning the two normative orientations, the conformity dimension was not related to deviant behaviours, nor to the voice constructs. Personal orientation towards conformity was also negatively related to cognitive flexibility, showing that individuals with high conformity scores are less cognitively flexible, and therefore less likely to generate ideas (Binard & Pohl, 2014). However, as expected, the rule adequacy, which is an orientation conceptually opposed to deviant behaviours (Dahling et al., 2012; Galperin, 2002), was negatively related to PSRB and constructive deviance. Furthermore, rule adequacy was only related to the prohibitive dimensions of voice, thus confirming a possible difference between proactive constructive behaviours and some positive deviant behaviours (Galperin, 2012).

Results showed good factorial stability and reliability of the NDPOS. However, according to Van de Schoot, Lugtig, and Hox (2012), it is common in social science to use questionnaire “to assess an underlying phenomenon with the goal to follow individuals over time or to compare groups” (p. 1). For this purpose, a scale should measure identical constructs with the same structure across different groups. The measurement of invariance allows one to test whether participants across all groups interpret individual questions, as well as the underlying latent factor, in the same way. Thus, we conducted invariance analysis on the NDPOS.

## Study 3 Scale Invariance Measurement

As the majority of the participants in study 1 and 2 were women, study 3 was conducted to control for whether the scale’s psychometric propriety could vary depending on gender. Morrison (2006) argued the importance of controlling for gender when performing studies related to deviant process. Indeed, women developed less rule-breaking behaviours and seemed to present lower risk-taking propensity and higher empathy than men. Furthermore, whistleblowing studies indicate a link between being a man and whistleblowing (Near & Miceli, 1985; MacNab, & Worthley, 2008), whereas the intent behind this practice is more related to being a woman (Liyanarachchi, & Newdick, 2009). These studies suggest that there may be a difference between men and women with respect to personal orientation to deviance and conformity.

### Method

Thus, to confirm that there were no variances in individuals’ responses according to gender, it was necessary to perform a study of invariance. We assessed the measurement invariance of the scale between males (N = 136) and females (N = 136) in a sample of 272 workers from the public (42%) and private sectors (58%), with an average age of 31.68 (SD = 10.85). This is considered an important issue for the psychometric development of tests (Brown, 2015) because it allows investigation of the extent to which different respondents interpret a given measure in a conceptually similar manner.

### Results

As a first step, we tested a CFA model for the male group (χ^2^ (48) = 62.05, p < .001; RMSEA = .04; CFI = .96; TLI = .95; SRMR = .06; AIC = 4360.005; BIC =4482.337) and the female group (χ^2^ (48) = 79.44, p < .001; RMSEA = .06; CFI = .94; TLI = .91; SRMR = .06; AIC = 4362.102; BIC = 4484.434). Subsequently, we tested for configural invariance by testing a multigroup CFA model in which we constrained the form, but not the loadings, intercepts and residuals of the model across the male and female group (Vandenberg & Lance, 2000). This model resulted in acceptable fit indices (χ^2^ (96) = 139.25, p < .001; RMSEA = .05; CFI = .95; TLI = .93; SRMR = .06; AIC = 8718.796; BIC = 9021.683). Next, we tested for metric invariance by putting an additional equality constraint on the factor loadings. Again, adding this constraint did not deteriorate model fit substantially (χ^2^ (104) = 151.78, p < .001; RMSEA = .05; CFI = .94; TLI = .93; SRMR = .07; AIC = 8716.483; BIC = 8990.524; Δχ^2^ = 12.572, Δdf = 8, p = ns). Finally, we tested for scalar invariance by also constraining the item intercepts to be equal across men and women. Similarly, fit indices were acceptable (χ^2^ (112) = 171.62, p < .001; RMSEA = .06; CFI = .93; TLI = .92; SRMR = .07; AIC = 8721.561; BIC = 8966.755; Δχ^2^ = 20.751, Δdf = 8, p < .05). However, the chi-square test showed significant difference between metric and scalar model, suggesting a pattern of non-invariance with the scalar model constrain. As the scalar model fit were worse than configural model and metric model, we conducted partial scalar invariance to estimate the entity of the non-invariance (Van de Schoot et al., 2012). We first relaxed the intercept that had the highest measure invariance (χ^2^ (111) = 170.55 p < .001; RMSEA = .06; CFI = .93; TLI = .92; SRMR = .07; AIC = 8722.568; BIC = 8971.368; Δχ^2^ = 2.23, Δdf = 1, p = ns). Second, as partial scalar invariance was observed (Edwards & Wirth, 2009), we tested partial residual invariance model (χ^2^ (122) = 174.981, p < .001; RMSEA = .05; CFI = .94; TLI = .93; SRMR = .08; AIC = 8707.216; BIC = 8916.353; Δχ^2^ = 21.15, Δdf = 10, p = ns). Compared to the male group, the female group tended to have a significantly lower mean factor score for deviant factors (ΔM = .216; p < .001) and higher scores for the normative factors (ΔM = .214; p < .001).

## General Discussion

The goal of this article was to propose a scale for normative and deviant personal orientations in the organisational context. To this end, we collected a large amount of information that allowed us to argue that the NDPOS satisfied the conditions necessary for construct validity. First, the EFA revealed that the best factor structure for the NDPOS was composed, as expected, of four factors. Second, following the CFA results, it appeared that the hypothesised four-factor model, composed of three items each, was the most suitable for measuring deviant and normative personal orientations. We reduced our initial pool of 20 items to a 12-item scale (Podsakoff & MacKenzie, 1994). Third, the correlation scores obtained for the two normative factors showed acceptable convergent validity with the conformity variable (Reysen & Branscombe, 2008), whereas the deviant factors were negatively correlated with it, confirming the difference between the concepts. Finally, invariance analysis showed that the scale could be used by both men and women; although, for a deviant equivalent situation, women tended to score lower than men. In summary, all the results obtained from the three studies conducted in this article suggest that the NDPOS is a valid measure for analysing personal orientation to deviance and normativity in a wide variety of organisational contexts.

### Limitations

Although the NDPOS showed good validity, some limitations were observed. First, all our measures were self-reported, and this approach may lead to common method bias issues (Podsakoff, MacKenzie, Lee, & Podsakoff, 2003). Although this method of collection is common (Ones, Viswesvaren, & Schmidt, 1993) and maintains the specificity of the deviant items (Dahling et al., 2012; Galperin, 2012), the NDPOS would benefit from being analysed also from the perspective of supervisors and collaborators (Robinson & O’Leary-Kelly, 1998). Second, the sample was essentially composed of women, and therefore it is necessary to investigate other samples that are better balanced for gender. To control this possible limitation, we added a factorial invariance test on a sample composed of an equal number of women and men. Women were less likely to develop deviant orientations than men, but the same scale structure emerged. Future research on deviance should focus on gender. Third, some of the convergent and divergent variables were behaviours tested according to the few orientation variables related to deviance and normativity indicated in the literature. It seems that some other orientation variables should have been tested for validity evidence. It would be useful for future research to engage in analyses showing that the developed scale incrementally predicts important outcomes over other similar concepts. Finally, the scale only makes it possible to measure its constitutive factors at a general level. The NDPOS does not permit targeting specific sectors and will require certain modifications depending on the environment. Thus, a third phase of validation with new behavioural and personal orientation elements would be necessary for more specific measurements.

### Future Research

In conclusion, this article, through the creation of a scale, makes it possible to demonstrate that there is a personal orientation component of deviance and normativity. Despite the limitations discussed above, the positive findings regarding discriminant, congruent, and convergent validity raise new questions and research paths. Indeed, the difference of correlation scores between the deviant behavioural variables and voice behaviour regarding the orientation towards rules adequacy makes it possible to suppose a conceptual difference between proactive and positive deviance behaviours. This difference must be analysed, as some authors (Vadera et al., 2013) incorporate behaviours such as voice (Van Dyne & Lepine, 1998) or taking charge (Morrison & Phelps, 1999) into the dimension of positive deviance. Thus, the NDPOS would make it possible to analyse how these behaviours are maintained with respect to deviance. Furthermore, future research should explore the role of normative and deviant personal orientation in the change and innovation process. For example, as it has been shown that insecurity at work affects innovative work behaviour (Niesen, Hootegem, Elst, Battistelli, & De Witte, 2018), it would be interesting to highlight the role played by deviant and normative personal orientation in this relationship. We suggest that there is a close link between the ability to deviate and innovative behaviour (e.g. Anderson, De Dreu, & Nijstad, 2004). This link is even clearer when one acts in response (or in prevision) to an organisational situation harmful to oneself or his or her colleagues or organisation (Anderson et al., 2004). Deviant and normative personal orientation would then be an essential antecedent to analyse in response to the call of researchers to determine the common antecedents of change and innovation-related behaviours (Potočnik & Anderson, 2016). The relationship with these orientations across different organisational levels of innovation should also be explored (see Battistelli, 2015). Indeed, a recent study showed that conformity affected the innovative process (Miron-Spektor, Erez, & Naveh, 2011); our scale should be useful in further exploring this relationship. Further research should investigate the relation of the NDPOS constructs to other constructs such as regulatory focus (Higgins, 1998) or felt responsibility (Morrison & Phelps, 1999). This might reveal a second-order factor between these variables, as Webster, Adams, and Beehr (2014) demonstrated in their research (*Core Work Evaluation*) on organisational commitment, work engagement, and job satisfaction. Finally, Moscovici (1979) defined deviance as a stage preceding change and the return to the norm by the creation of a minority group. This dynamic aspect will benefit from investigation of the role of deviant and normative personal orientations in the development of constructive and destructive organisational behaviours.
